# Detection of Performance of Hybrid Rice Pot-Tray Sowing Utilizing Machine Vision and Machine Learning Approach

**DOI:** 10.3390/s19235332

**Published:** 2019-12-03

**Authors:** Wenhao Dong, Xu Ma, Hongwei Li, Suiyan Tan, Linjie Guo

**Affiliations:** 1 College of Engineering, South China Agricultural University, Guangzhou 510642, China; 2 College of Electronic Engineering, South China Agricultural University, Guangzhou 510642, China

**Keywords:** machine vision, color model, seeding performance, segmentation, machine learning

## Abstract

Monitoring the performance of hybrid rice seeding is very important for the seedling production line to adjust the sowing amount of the seeding device. The objective of this paper was to develop a system for the real-time online monitoring of the performance of hybrid rice seeding based on embedded machine vision and machine learning technology. The embedded detection system captured images of pot trays that passed under the illuminant cabinet installed in the seedling production line. This paper proposed an algorithm for fixed threshold segmentation by analyzing the images with the exploratory analysis method. With the algorithm, the grid image and seed image were extracted from the pot tray image. The paper also proposed a method for obtaining pixel coordinates of gridlines from the grid image. Binary images of seeds were divided into small pieces, according to the pixel coordinates of gridlines. Each piece corresponded to a cell on the pot tray. By scanning the contours in each piece of the image to check whether there were seeds in the cell, the number of empty cells was counted and then used to calculate the missing rate of hybrid rice seeding. The seed number sowed in pot trays was monitored while using the machine learning approach. The experimental results demonstrated that it would consume 4.863 s for the device to process an image, which allowed for the detection of the missing rate and seed number in real-time at the rate of 500 trays per hour (7.2 s per tray). The average accuracy of the detection of missing rates of a seedling production line was 94.67%. The average accuracy of the detection of seed numbers was 95.68%.

## 1. Introduction

Hybrid rice is an important grain crop in China. Most of the hybrid rice is transplanted while using nursery transplanting techniques. Seedlings are produced on rice seedling production lines in order to improve the work efficiency. Hybrid rice has a strong tillering ability, which can increase the number of effective panicles. A lower-cost and sparse-planting agronomic practice was required because of its strong tillering ability and the high cost of hybrid rice seed. One to three grains are usually sown in each tray cell in order to achieve high quality and high yield in the mechanized cultivation of hybrid rice [1,2]. This will achieve efficient utilization of the seeds and space. Due to the low sowing rate, there will be some tray cells that are not sown. This has an adverse effect on the final yield of hybrid rice. Therefore, it is important to detect the performance of hybrid rice seeding in real time, so as to adjust the seeding device in time and improve the performance of the rice seedling production line.

At present, studies are focused on the detection and evaluation of seeding performance while using one of the following methods: traditional, photoelectric, high-speed camera, and computer vision methods. The traditional method uses human eyes to evaluate the seeding performance. The workload is heavy and it is very inefficient. It is not suitable for the automation of agricultural machinery. Researchers have used photoelectric sensors to test the performance of seeders. Jia [3] used photoelectric sensors to detect the performance of an air-suction metering device. Kocher [4] used photoelectric sensors to quickly evaluate the uniformity of the seed spacing. Photoelectric sensors are widely used to detect the seeding leakage and replay precision seeders due to their low cost, but they are not suitable for detecting the performance of seeders from which a large amount of seeds continuously fall down.

With the development of computer vision and image processing technology, they are widely used in agriculture. Some studies [5,6] used machine vision to evaluate and classify the quality of seeds and rice. Leemans [7] proposed a method for guiding the precision seed drill while using machine vision. Research regarding noncontact measurement and grain counting using machine vision and image processing technology has been widely carried out. Kim [8] used machine vision technology to detect the embryonic orientation of Cucurbitaceae seeds when they were planted in a pot tray. Zhang [9] developed an ellipse-fitting algorithm to separate the touching grain kernels in images. Machine vision was also used to detect the performance of hybrid rice seeding. Qi [10] used machine vision and LabVIEW software to realize the detection of seeding cavities in hybrid rice seeding. Tan [11,12,13] used machine vision and MATLAB to detect the performance of hybrid rice seeding. Most of the studies using machine vision mentioned above were based on the Windows platform. The cost of the detection systems that were used in the research is high, and the size of the systems is large. Accordingly, they are mostly used in the laboratory and they cannot be integrated with production facilities. Additionally, they cannot be used for online detection in actual production at facilities.

With the development of embedded technology, combining machine vision technology and embedded equipment in agricultural machinery has been a growing trend. Embedded equipment has features, such as small size, low power consumption, ease of carrying, and ease of integrating into existing facilities. Embedded devices are increasingly used in agricultural machinery. Some researchers [14,15] developed systems for measuring the plant leaf area based on the Android platform while using smartphone cameras. Tu [16] developed a system for classifying pepper seeds based on the Android platform. Ma [17] developed a visual measurement and portable instrumentation method for crop seed phenotyping. Tan [18] designed a system for catching seed images and sending the images to a computer through wireless transmission while using an embedded Linux operating system (OS) and machine vision. With the improvement and development of an open source cross-platform computer vision library, it becomes more convenient and much easier to combine an embedded system with machine vision to develop complex image processing applications. These platforms and techniques provide technical support for the development of agricultural machinery toward automation and intellectualization.

A system for detecting performance of hybrid rice seeding with pot trays was developed based on embedded Linux OS and OpenCV, which is an open-source and cross-platform computer vision library, and combining an embedded system with machine vision technology and machine learning approach. A laboratory experimental was carried out. The performance of the system was evaluated in terms of accuracy and efficiency.

## 2. Materials and Methods

### 2.1. Device and Tools

**Figure 1:**
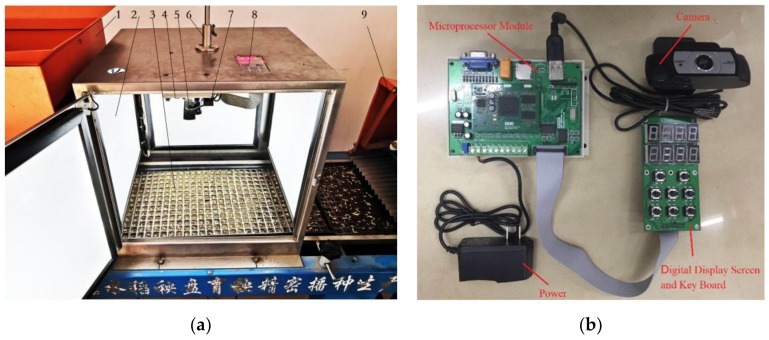
Diagram of detection device. (**a**) Detection device on seedling production line: (1) soil spreading device, (2) lighting board, (3) pot tray, (4) microprocessor module, (5) camera, (6) fixture adjusting rod, (7) camera fixture, (8) digital display screen and keyboard, and (9) seeding device. (**b**) Embedded system of the device.

The device that is used to detect the performance of hybrid rice seeding is installed on the 2ZSB-500 rice seedling production line at South China Agricultural University. It is located between the seeding device and the spreading soil device, as shown in Figure 1a. The detecting device, which is based on embedded machine vision technology, consists of an illuminant cabinet and an embedded system. The embedded system is composed of a microprocessor module, a high-definition camera, a keyboard, and a digital display screen. The illuminant cabinet is mainly composed of a metal box, four lighting boards, a camera fixture, and a fixture adjusting rod.

Figure 1a shows the inside of the cabinet. A lighting board is installed on each wall of the metal box, which is composed of some LED beads, a light guide board, and a light leveling board. Light guide boards and light leveling boards can distribute the light that is emitted by LED beads evenly inside the illuminant cabinet. The brightness regulator can adjust the brightness of the lighting board, so that the light intensity in the cabinet can meet the needs of image acquisition. The microprocessor module is fixed on the inner side of the upper wall of the metal box, the keyboard and LED display screen are fixed on the outside of the panel above the metal box, and the UVC high-definition camera is installed on the camera fixture. Pushing or pulling the fixture adjusting rod can change the distance between the camera and pot trays. By rotating the rod, the angle of the camera on the fixture can be adjusted. There is a U-shaped dovetail groove on the camera fixture in which the camera can glide. The groove makes it possible to adjust the position of the camera precisely. Thus, the camera can be accurately fixed in positions, where it can catch images of predetermined areas of pot trays, with the fixture adjusting rod and the groove on the fixture.

The embedded system consists of a microprocessor module, UVC high-definition camera, keyboard, and LED display module, as shown in Figure 1b. A low-power microprocessor that is based on ARM CortexTM-A8 core is used as the CPU of the microprocessor module (model S5PV210AH, Samsung). There is 256 M dynamic random access memory (DRAM) and 128 MB NAND Flash, which is the program and data memory of the module. The camera that is used in the system is a high-definition webcam (model C920, Logitech). The program in the embedded system was developed with C++ programming language and it is based on an embedded Linux operating system, which is open source. It is programmed with OpenCV, which is a cross-platform computer vision library. The OpenCV library that is used in this paper is compiled on Ubuntu with arm-linux-gcc4.4.3, and its version is 3.0.

### 2.2. Algorithm for Seeding Performance Detection

**Figure 2:**
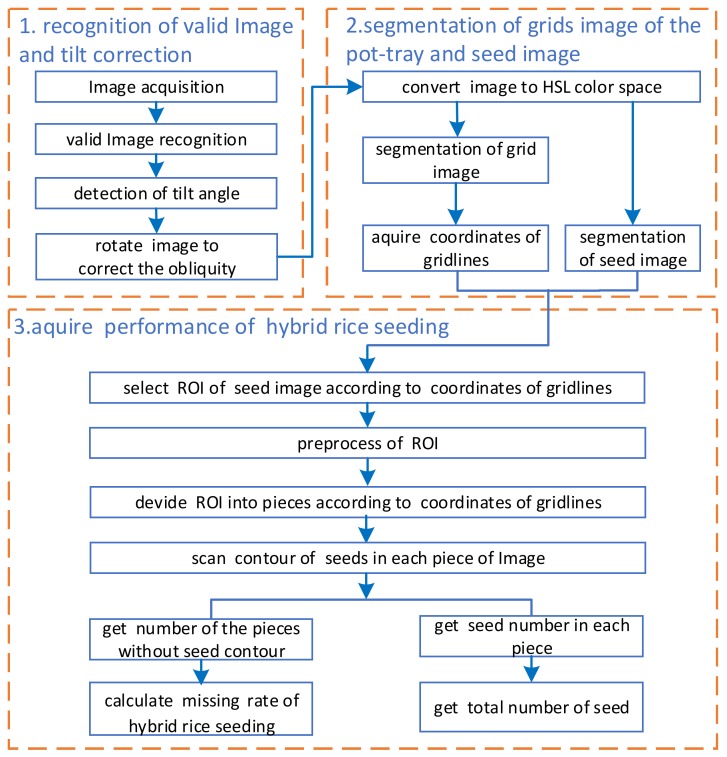
Flowchart of detection of performance of hybrid rice seeding in pot tray. HSL, hue, saturation, lightness; ROI, region of interest.

The pot tray passes under the illuminant cabinet after the seeds are sown on a pot tray. While the pot tray is passing by, the embedded system will capture its image to detect the performance of hybrid rice seeding. Figure 2 shows the flowchart of detecting the performance of hybrid rice seeding in pot trays.

#### 2.2.1. Recognition of Valid Images

**Figure 3:**
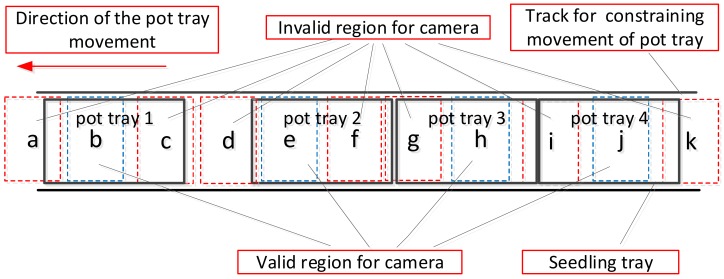
Sketch of pot trays moving on seedling production line.

There are several work steps on the rice seedling production line, such as spreading subsoil, compacting subsoil, seeding and spreading topsoil, and so on. Pot trays are transported to the positions of the work steps one by one on the conveyor belt in the production of seedlings. Both sides of the conveyor belt are installed with a track to constrain the movement of the pot trays deviating from the belt, as shown in Figure 3.

While the detection device is working, it captures images of pot trays passing by continuously. Real performance of seeding cannot be obtained from images that include the beginning or end of a pot tray. Thus, an image will be regarded as invalid when it contains a beginning or end of a pot tray (shown as regions a, c, d, g, i, and k in Figure 3); otherwise, it will be regarded as a valid image (shown as regions b, e, h, and j in Figure 3). Only the first image without the ends of the tray will be regarded as valid to ensure that a pot tray will be detected only once. Therefore, region f in Figure 3 is also regarded as an invalid image. Invalid images will be discarded, and they will not be further processed.

**Figure 4:**
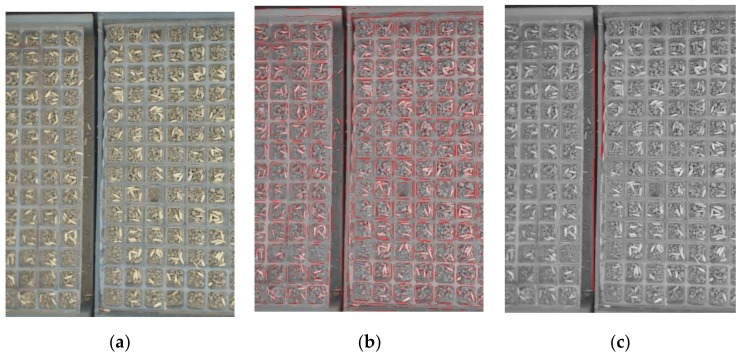
Process to detect beginning or end area of pot tray: (**a**) image with beginnings and ends of pot trays; (**b**) lines detected with line segment detector (LSD); and, (**c**) lines left after being filtered.

Figure 4 shows the process of recognizing the beginning or end of a pot tray. Figure 4a is an image that was captured on the seedling production line. There are beginnings and ends of pot trays in the image. In this paper, a line segment detector (LSD) was used to detect the beginning or end of a pot tray. After an image was converted to a gray image, all of the line segments in the gray image were detected with LSD, as shown in Figure 4b. Subsequently, a vertical projected length of 100 pixels was set as a threshold to filter the line segments. After the line segments were filtered, only line segments whose vertical projected length was over 100 pixels were left, as shown in Figure 4c. All of the short line segments, which were composed of the contours of seeds or soil particles, and line segments, composed of the edges of both sides of pot trays, were removed. After that, there were still line segments left, which showed that the beginning or end of a pot tray was captured.

#### 2.2.2. Image Tilt Angle Detection and Correction 

**Figure 5:**
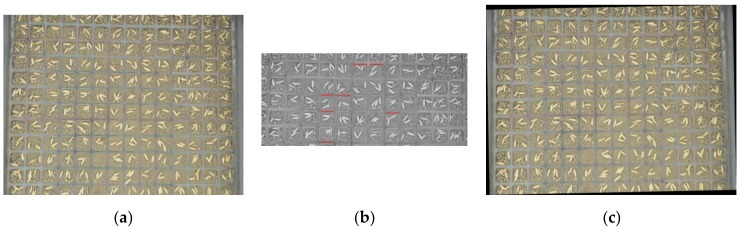
Process of image obliquity correction: (**a**) original image; (**b**) lines detected with LSD; and, (**c**) rotated image.

Images of pot trays collected on the rice seedling production line are a little tilted when compared to the horizontal direction, as shown in Figure 5a. It is necessary to correct the obliquity of the images to obtain accurate coordinates of gridlines of a pot tray. In this paper, LSD is also used to detect the horizontal line segments, which are horizontal borders of the cells on a pot tray. The degree of obliquity of an image is calculated according to the slope of the detected line segments. Figure 5 shows the process to correct the obliquity of an image.

#### Detection of Image Obliquity

A part instead of the whole image is selected in order to reduce the time to detect the obliquity of an image, as shown in Figure 5b. First, a region of (7,070,800,500) is selected as the region of interest (ROI) of the image, and the ROI is then converted to a gray image. LSD is used to detect all the line segments in the ROI, and then a horizontal projected length of 45 pixels is set as a threshold to filter the line segments. After being filtered, only the line segments that are part of the horizontal gridlines and longer than 45 pixels are left, as shown as the red lines in Figure 5b. The slopes of all remaining line segments are calculated while using the coordinates of the two endpoints of the line segments. The average value of all slopes of the line segments is calculated after removing the maximum and minimum values among the slopes to reduce the error of obliquity detection. The formula for calculating the slope of a line segment is as follows:

(1) $$k=\frac{y_{2}-y_{1}}{x_{2}-x_{1}}$$

where $x_{1}$ and $x_{2}$ are the *x*-coordinates and $y_{1}$ and $y_{2}$ are the *y*-coordinates of the two endpoints of the line segment, and *k* is the slope of the line segment; and,

(2) $$K=\frac{k_{1}+k_{2}+\ldots +k_{n}-k_{max}-k_{min}}{n-2}$$

where n is the number of line segments, $k_{1}–k_{n}$ are the slopes of the line segments, $k_{max}$ is the maximum value among $k_{1}–k_{n}$, $k_{min}$ is the minimum value among $k_{1}–k_{n}$, and K is the average value of the slopes.

While using the average value of the slopes, the tilt angle of an image is obtained. The formula for calculating the tilt angle of an image is shown, as follows:

(3) $$\theta =\frac{K\pi }{180}$$

where θ is the tilt angle of the image.

#### Image Tilt Correction

It is necessary to rotate the image to correct the obliquity when the tilt angle of the image is detected. After rotation, the gridlines of the pot tray are absolutely horizontal or vertical, as shown in Figure 5c. In this paper, the images are rotated through affine transformation. The center of the image is selected as the point around which the image is rotated. Its x- and y-coordinates are as follows:

(4) $$\left\{\begin{array}{c} centre.x=\frac{width}{2}\\ centre.y=\frac{height}{2} \end{array}\right.$$

where width is the width of an image and height is the height of the image.

The affine transformation matrix A is as follows:

(5) α=cosθ

(6) β=sinθ

where θ is the tilt angle of the image.

(7) $$A=\left[\begin{array}{lll} \alpha & \beta & \left(1-\alpha \right)\times centre.x-\beta \times centre.y\\ -\beta & \alpha & \beta \times centre.x+\left(1-\alpha \right)\times centre.y \end{array}\right]$$

With matrix A, the image undergoes affine transformation to complete the image rotation correction.

#### 2.2.3. Segmentation of Grid and Seed Images

##### Analysis of Different Regions of the Image

**Figure 6:**
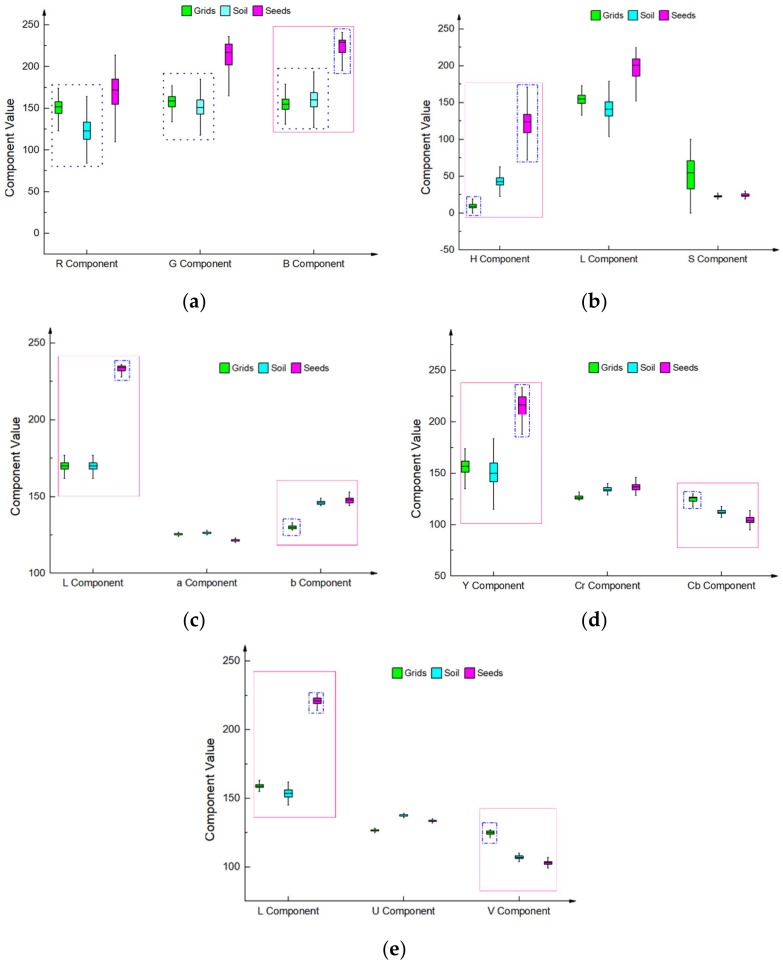
Various color component distribution graphs of grids, seeds, and soil using five color models: (**a**) red, green, blue (RGB) model; (**b**) HSL model; (**c**) Lab model; (**d**) YCrCb model; and, (**e**) LUV model.

Seed and grid images of the pot tray must be segmented from the captured image in order to detect the missing rate of hybrid rice seeding in pot trays on the precision seedling production line. Image regions of seeds, grids, and soil were selected to conduct a comparative analysis of different color models (red, green, blue (RGB); hue, saturation, and lightness (HSL); Lab; YCrCb; and, LUV). A total of 20 images were selected for analysis. The seed, grid, and soil pixels were artificially obtained from each image with 300 pixels for each part. A total of 18,000 pixels were chosen to create the box plots in Figure 6.

As shown in Figure 6a, the three components values of grid and soil overlap each other in the RGB color model, as shown in the black rectangles. Therefore, it is impossible to segment the grid images from images in the RGB color model. Only seed images can be segmented while using the B component value, as shown in the blue rectangle. From Figure 6b–e, it is found that seed and grid images can both be segmented in the other color models (HSL, Lab, YCrCb, and LUV). Therefore, the captured image must be converted to a color model other than RGB in order to segment seed and grid images. The application programming interface (API) function ‘cvtColor’ in the library of OpenCV is used to convert RGB Images to other color models. Using different parameters, the RGB images can be convert to different color models using the same function. Table 1 shows the time to convert the RGB model to the other color models.

As shown in Table 1, the time to convert images from RGB to HSL is much less than that of the other models. Therefore, the HSL color model was selected for image segmentation in order to shorten the total time for image processing as much as possible.

Table 2 shows the distribution of color component values of grids, soil, and seeds in the HSL color model.

**Figure 7:**
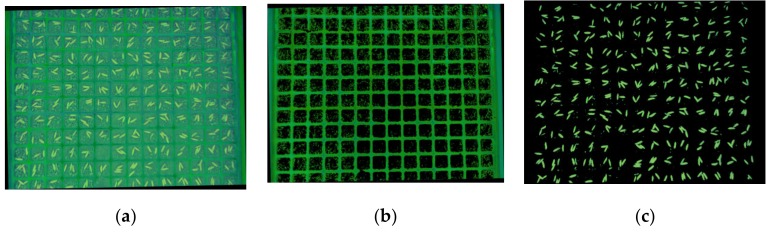
Result of image segmentation: (**a**) image in HSL color model; (**b**) grid image segmented; and, (**c**) seed image segmented.

Table 2 shows that there is little overlap in the distribution of H component values of grid, soil, and seed images. A fixed threshold segmentation method can be used to segment the grid and seed images using the H color component value. Figure 7 shows the segmentation result.

##### Steps for Fixed Threshold Segmentation

The algorithm to segment seed and grid images from an HSL image is described, as follows:

Step 1. Convert the RGB image to the HSL color model, as shown in Figure 7a.

Step 2. Set maximum and minimum thresholds for the H component value, and mark them as H_max and H_min, respectively.

Step 3. Obtain the mask image. First, a triple-channel image, which has the same size as the HSL image obtained in step 1, is created as a mask image. Afterwards, set the value of each pixel in the mask image to 0 to initialize it. Finally, the H component value of each pixel (marked as H_value) in the HSL image is compared with H_max and H_min. If the H_value is larger than H_min and smaller than H_max, the value of the corresponding pixel in the mask image is set to 1. 

Step 4. Segment image. Using "and" operation between the HSL image and the mask image, the result of the operation is the segmented image.

The grid and seed images are extracted from the HSL image while using different thresholds, as shown in Figure 7b,c.

Photoshop is used to segment the seed image from the rotated image and erase the noise points in the seed image, and the image is then converted to a binary image. This image can provide criteria to evaluate the algorithm of segmentation proposed in this paper. The seed image that is segmented by a fixed threshold is converted to a binary image, and its pixel sum is compared with that of the image used for the criteria. 20 images were selected to be evaluated using the same method, and the average accuracy rate for segmentation was 99.45%.

#### 2.2.4. Obtaining Pixel Coordinates of Gridlines 

**Figure 8:**
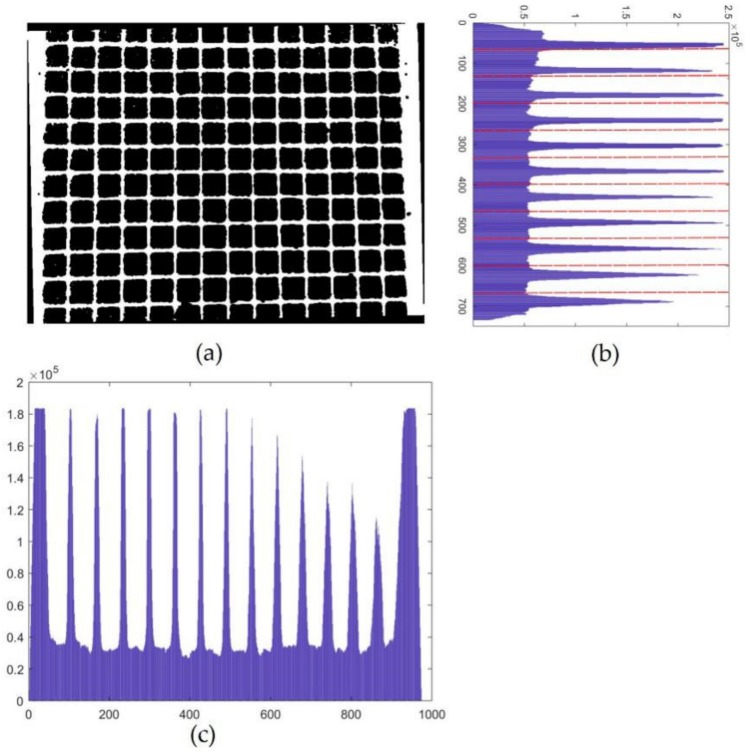
Schematic diagram of pixel projection: (**a**) binary image of grids; (**b**) histogram of rows’ pixel sums; and, (**c**) histogram of columns’ pixel sums.

The algorithm to get pixel coordinates of gridlines is described, as follows:

Step 1. Preprocess the grid image. First the grid image is converted to a gray image, then the gray image is converted to a binary image with the Otsu method [19]. Finally, the noise pixels in the binary image are removed with the morphology noise reduction method. Thus, a relatively pure binary image of the grid was obtained, as shown in Figure 8a.

Step 2. Get the pixel sum of every row and every column of the binary image. Add the values of pixels in a row to get the pixel sum of the row, and record the row number and pixel sum as a pair of data. Assuming that there are m columns and n rows in the binary image, and then n pairs of data can be obtained. Figure 8b shows a histogram of row numbers and their pixel sums. In the histogram of the row pixel sum, a regulation is found that the pixel sums of the rows in which there are horizontal gridlines are much bigger than those of rows in which there is no horizontal gridline. A histogram of column numbers and their pixel sums is drawn while using the same method, as shown in Figure 8c. 

Step 3. Obtain the pixel coordinates of gridlines. Divide the n pairs of data (row numbers and pixel sums) into 11 groups by average, being shown as the dashed red lines in Figure 8b. Subsequently, query the maximum value of the pixel sum in every group and obtain its corresponding row number. The row number is regarded as the pixel coordinate of a horizontal gridline. Pixel coordinates of vertical gridlines can be obtained with the same method.

#### 2.2.5. ROI Selection of Seed Images

**Figure 9:**
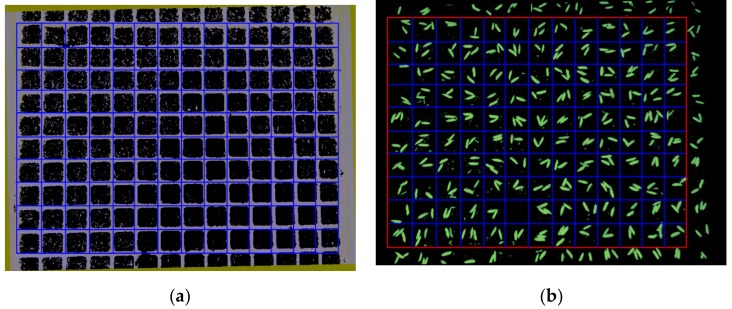
(**a**) Lines drawn in grid image; and, (**b**) ROI of seed image.

Drawing row and column lines using their pixel coordinates on the grid image, as shown in Figure 9a. It shows that the blue grids that are drawn coincide with the grids in the grid image. A region including 10 rows and 13 columns is selected as the ROI of the seed image because there is always a region that includes a complete 10 rows and 13 columns of cells in the shooting area of the camera, shown as the red rectangle in Figure 9b.

#### 2.2.6. Preprocessing the ROI

**Figure 10:**
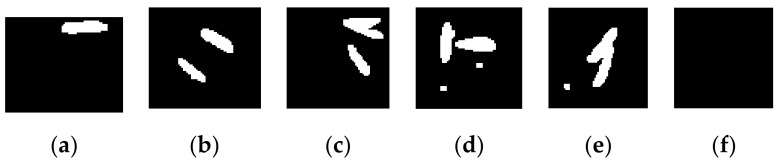
Pieces of seed image being split according to grids: (**a**–**c**) pieces with seeds; (**d**,**e**) pieces with noise pixels; and, (**f**) piece without seeds.

First, the ROI of a seed image is processed in the same way as the grid image is preprocessed described above. Subsequently, the image of the ROI is divided into 130 pieces according to the gridline coordinates. Each piece of the image corresponds to a hole on the pot tray. Figure 10 shows parts of the pieces. After noise reduction, the small noises are removed, but there are still many bigger noise points, composed of soil particles or impurities in the soil, shown in Figure 10d,e.

#### 2.2.7. Detection of the Missing Rate 

**Figure 11:**
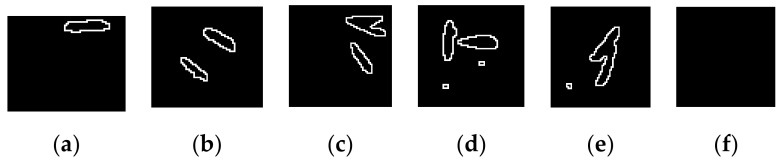
Contours in pieces of the image: (**a**–**c**) pieces with seeds; (**d**,**e**) pieces with noise pixels; and, (**f**) piece without seeds.

Step 1. Scan the contours in each piece of the image. Figure 11 shows parts of contours. If there is no seed in a piece of the image, there will not be any contours in it, as shown in Figure 11f.

The steps for detecting the missing rate of hybrid rice seeding is described as follows:

Step 2. Filter small contours in some pieces of images. It is necessary to remove the small contours or they will be mistaken for seeds because there are small contours that are composed of soil particles or impurities in the soil, as shown in Figure 11d,e. Check the area of every contour in a piece of image. The area of every contour is obtained using the API function ‘contourArea’ in the library of OpenCV. The normal size of a seed contour is larger than 30 pixels and the area of an overwhelming majority of impurity contours are smaller than 30 pixels. Therefore, an area of 30 pixels is set as a threshold to filter the contour of impurities. The contour will be removed if the area of a contour is smaller than 30 pixels. 

Step 3. Count the number of pieces in which there are no contours to get the number of cells in which there are no seeds, and calculate the missing rate, as follows:

(8) $$\mathrm{Missing}\;\mathrm{Rate}=\frac{m}{N}\times 100\%$$

where *m* is the number of empty cells and *N* is the total number of cells.

#### 2.2.8. Detection of the Seed Number

Machine learning approaches are used to detect seed number in the ROI. The algorithm to detect the seed number is described, as follows:

Step 1. Generating a BP (back propagation) neural network. A software tool that was developed with Visual Studio 2012 and OpenCV3.0 establishes a BP neural network. The BP neural network has an input layer, a hidden layer, and an output layer. The input layer has three nodes, the hidden layer has six nodes, and the output layer has one node. The activation function of the BP neural network is as follows:

(9) $$f\left(x\right)=\frac{1-e^{-x}}{1+e^{-x}}$$

Step 2. Training and saving the BP neural network. The area, perimeter, and shape factor of a contour are chosen as the node parameters of the input layer. The seed number in a contour is chosen as the node parameter of the output layer. The perimeter of contours can be obtained while using API function ‘arclength’ in OpenCV library. The formula for calculating the shape factor is as follows:

(10) $$F=\frac{C^{2}}{4\pi A}$$

where *F* is shape factor of seed contours, *C* is perimeter of seed contours, and *A* is area of seed contours.

Select some contours as training samples. Using area, perimeter, and shape factor of the contours as input parameters and seed number in the contours as the output result to train the BP neural network.

Table 3 shows the number of the training samples, where Type 1 represents that there is one seed in each contour sample, Type 2 represents that there are two seeds in each contour sample, Type 3 represents that there are three seeds in each contour sample, Type 4 represents that there are four seeds in each contour sample, and Type 5 represents there are more than four seeds in each contour sample.

After the BP neural network is trained and produced, it is saved in XML (extensible markup language) format in the embedded detection system.

Step 3. Prediction of seed number. The BP neural network is loaded when the embedded detection system is started. The area, perimeter, and shape factor of each contour are obtained and inputted into the BP neural network to predict the seed number in each contour. Add seed number in each contour together, the total seed number in the ROI can be obtained.

## 3. Results and Discussions

The test was done on a rice seedling production line. The hybrid rice species used in the test was WuYou 1179 and its thousand grain weight was 25 g. The seedling tray used in the test was a soft pot tray, with a size of about 581 × 284 mm (length × width). There were 29 rows and 24 columns on a pot tray and a total of 406 cells. The productivity of the rice seedling production line was set at 500 trays per hour. The system’s camera can capture an image that includes 10 rows and 13 columns of a pot tray, with a resolution of 960 × 720 pixels.

The images of pot trays were captured when the conveyor belt is in motion. The speed of the conveyor belt is about 0.081 m/s. The images that were captured without the strong light of the illuminant cabinet are not clear. Accordingly, the illuminant cabinet is indispensable for the detection system. Clear photos of pot trays were obtained with the assisting of the illuminant cabinet. The illuminance of the light in the illuminant cabinet was about 3920 LUX.

### 3.1. Performance of Missing Rate Detection

An image of every pot tray passing under the illuminant cabinet was collected, and an ROI, including 10 rows and 13 columns (130 cells), was selected as a detection object. The cells at the top and bottom of the images may be incomplete, because they are not in the complete view of the camera. Therefore, the cells at the top and bottom of the images are not selected as ROI, and the selected ROI is representative. 

The number of empty cells in each detection was recorded. The total cells in three detections was 390, which is close to the total number of cells on a pot tray (406 cells). Thus, we could calculate the missing rate every three successive detections to evaluate the seeding performance. Table 4 shows twenty consecutive statistical results.

Table 4 shows that the average accuracy of detection of missing rate of hybrid rice seeding is 94.67%. The formula for calculating the relative error of detection is shown, as follows:

(11) $$\delta =\;\frac{\left|\;v\;\prime \;-v\right|}{v}\times 100\%$$

where δ is the relative error of detection, v^’^ is the average value of system measurement, and v is the average value of manual measurement.

### 3.2. Performance of Seed Number Detection

BP neural network was used to detect seed number in ROI. The seed numbers in every three consecutive images were added together, and the result is regarded as detection. The relative error of twenty detections is calculated while using formula 11. The average accuracy of detection of seed number is 95.68%.

### 3.3. Discussion

By analyzing the images, we found reasons for error detection, as follows:

(1) There were some mildewed seeds in the experiment, and their color was very similar to the soil on the background. This made it difficult to use the fixed segment algorithm to completely segment the mildewed seeds from the image. Only small partial images of mildewed seeds could be segmented. The contour area of partial seeds was very small. The contour was eventually removed due to its small size when filtering. This resulted in the detected number of holes without the seeds being larger than that of a real situation.
(2) There were big soil particles in the cells and the particles covered parts of seeds in the cells. Only small parts of seeds that were not covered by soil particles were captured by the camera. The contour area of these parts was so small that it was removed when the contours were filtered, which resulted in the number of empty cells being larger than it should be.
(3) There were very few impurities in the subsoil, whose color is similar to that of the seeds. Some were big enough to be mistaken as parts of seeds, which decreased the number of empty cells, and resulted in a smaller missing rate.

It is necessary to pick out the mildewed seeds and the soil should be sieved with a fine sieve to decrease the detection error and ensure the quality of seedlings. It is not easy to completely extract seed if the color of the background is similar to the seed color. Therefore, further study should be carried out while using deep learning approaches to detect the performance of seeding.

## 4. Conclusions

The conclusions of this study are as follows:

(1) An embedded seeding performance detection system was developed while using the embedded technology and machine vision technique. The proposed system can be integrated into the rice seedling nursery production line to evaluate the seeding performance on the go.
(2) The component values of different parts of the image were analyses using different color models. A fixed threshold segmentation method was proposed based on the HSL model. The grid and seed images were extracted while using the proposed method with an average segmentation accuracy of 99.45%.
(3) An algorithm was developed for calculating the missing rate of the seedling production line. The detection accuracy was 93.33% with an average processing time of 4.863 s, which was lower than the tray passing time of 7.2 s at a production rate of 500 trays per hour. This enabled the detection to be a real-time operation.
(4) The number of seeds was also measured while using a machine learning approach and the average accuracy was 95.68%.

## Figures and Tables

**Table 1:** Time for segmentation in different color models.

Color Space	HSL	Lab	YCrCb	LUV
Time to convert (ms)	191	612	598	602

**Table 2:** Component values of grids, seeds, and soil in HSL color model.

Component of Different Parts	Color Component		
	H	S	L
Component value of grids	3–30	10–88	108–173
Component value of soil	28–68	17–28	83–187
Component value of seeds	70–168	18–31	153–232

**Table 3:** Sample type and sample number.

Contour Type	Type 1	Type 2	Type 3	Type 4	Type 5
Sample number	2280	1216	822	620	586

**Table 4:** Statistical results for missing rate.

Code	Total Cells	Manual Measurement of Empty Cells	System Measurement of Empty Cells	Manual Measurement of Missing Rate (%)	System Measurement of Missing Rate (%)	Relative Error (%)
1	390	10	11	2.56	2.82	10.00
2	390	9	10	2.31	2.56	11.11
3	390	8	8	2.05	2.05	0.00
4	390	7	7	1.79	1.79	0.00
5	390	10	11	2.56	2.82	10.00
6	390	9	10	2.31	2.56	11.11
7	390	6	6	1.54	1.54	0.00
8	390	5	5	1.28	1.28	0.00
9	390	9	10	2.31	2.56	11.11
10	390	4	4	1.03	1.03	0.00
11	390	8	9	2.05	2.31	12.50
12	390	3	3	0.77	0.77	0.00
13	390	6	6	1.54	1.54	0.00
14	390	7	7	1.79	1.79	0.00
15	390	10	11	2.56	2.82	10.00
16	390	11	12	2.82	3.08	9.09
17	390	9	10	2.31	2.56	11.11
18	390	7	7	1.79	1.79	0.00
19	390	5	4	1.28	1.03	20.00
20	390	7	7	1.79	1.79	0.00
Average	390	7.5	7.95	1.92	2.03	5.33
